# Hard on Dr. Atkinson

**Published:** 1881-09

**Authors:** 


### EDITORIAL, ETC.

*Hard on Dr. Atkinson.*—The bright and cheery editor of the *Ohio State Journal of Dental Science,* than which no more pleasant periodical visits us, says of that very extraordinary report of Dr. Atkinson on "Dental Literature and Nomenclature," made to the American Dental Association of 1880:

"The tendency of this report is to greatly elevate the standing of our profession, for if it is adopted, and it is to be understood and used, the course of dental study will surpass in degree, and in the time required to master it, the *curriculi* of medicine, law, theology, literature and politics, with Chinese, Choctaw and legerdemain added. The document is simply amazing, and is deeper than anything known or unknown, except the bottomless pit." * * * "But after all there is this redeeming feature; the man who works on such reports, year by year, is not doing anything worse. We know a man, slightly deranged, who could be kept quiet and docile, months at a time, simply by furnishing him tools and timber to construct a perpetual motion."

